# Peritumoral habitat radiomics predicts axillary lymph node metastasis in breast cancer

**DOI:** 10.1016/j.isci.2026.115365

**Published:** 2026-03-16

**Authors:** Jingwen Ding, Zhikun Qiu, Hongbin Peng, Shuyuan Chen, Qiaowei Zhong, Yideng Zhang, Zhiwei Yang, Zhenfeng Huang

**Affiliations:** 1 The Fifth Affiliated Hospital, Sun Yat-sen University, Zhuhai 519000, China; 2 Department of Breast Surgery, Huizhou Central People’s Hospital, Huizhou 516000, China; 3 Linyi People’s Hospital, Linyi 276000, China; 4 Zhuhai Campus of Zunyi Medical University, Zhuhai 519041, China

**Keywords:** Artificial intelligence applications, Medical imaging, Oncology

## Abstract

Accurate preoperative prediction of axillary lymph node metastasis (ALNM) is critical for personalized management of breast cancer. Here, we developed a habitat-aware, multiscale radiomics framework based on dynamic contrast-enhanced MRI to characterize intratumoral heterogeneity and peritumoral microenvironmental changes. Intratumoral habitats were delineated using superpixel segmentation and clustering, and radiomic features from habitats, concentric peritumoral regions, and clinical variables were integrated to construct predictive models. A fusion model combining the habitat signature, 1-mm peritumoral signature, and clinical factors achieved the best performance, consistently outperforming conventional radiomics approaches, with AUCs of 0.940, 0.905, 0.857, and 0.884 in the training, validation, and two independent test cohorts, respectively. These findings demonstrate that habitat-guided multiscale radiomics enables accurate, interpretable, and noninvasive assessment of nodal metastasis risk and may support individualized surgical and therapeutic decision-making in breast cancer.

## Highlights

• Explainable DCE MRI radiomics predicts axillary lymph node metastasis
• Habitat and peritumoral features quantify tumor heterogeneity across scales
• The combined model achieves high accuracy across independent cohorts
• Provides a noninvasive tool to guide personalized surgical decisions

## Graphical abstract

**Figure:**
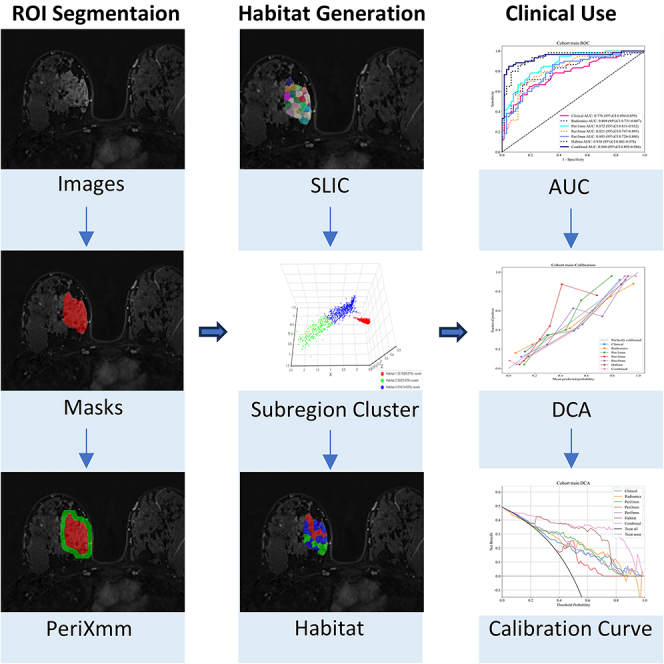

## Introduction

Breast cancer remains a global health burden and is the most commonly diagnosed malignancy among women worldwide.^1^ Axillary lymph node metastasis (ALNM) serves as a critical prognostic indicator and plays a pivotal role in guiding treatment decisions.^2^^,^^3^ Patients with nodal involvement typically face significantly poorer outcomes. Therefore, accurate preoperative assessment of ALNM is essential for personalized treatment planning, enabling appropriate surgical and systemic interventions while minimizing unnecessary invasive procedures.^4^

Current clinical practice relies on conventional imaging and surgical methods to evaluate axillary status. Although widely used, non-invasive imaging modalities such as ultrasound and magnetic resonance imaging (MRI) have limited diagnostic performance. MRI can detect only approximately 10% of lymph node micrometastases in early breast cancer.^2^ While sentinel lymph node biopsy (SLNB) remains the gold standard due to its high accuracy, it is an invasive procedure associated with complications such as lymphedema, nerve injury, and increased healthcare costs.^5^^,^^6^ This trade-off between diagnostic precision and procedural morbidity underscores the pressing need for accurate, non-invasive tools to predict ALNM and optimize patient selection for SLNB.^7^

Radiomic analysis of dynamic contrast-enhanced (DCE) MRI has emerged as a promising non-invasive approach to this challenge.^8^ Radiomics enables the extraction of high-dimensional quantitative features that reflect tumor phenotype, heterogeneity, and underlying biology. Prior studies have demonstrated that DCE-MRI-derived radiomic signatures, particularly when combined with clinical variables and molecular subtypes, can achieve robust predictive performance for ALNM, with AUC ranging from 0.86 to 0.91 in multi-center validation cohorts.^9^ However, many of these high-performing signatures are based on conventional whole-tumor segmentation, which averages spatial heterogeneity and may obscure biologically distinct subregions critical for metastasis.^10^ Similarly, while the peritumoral microenvironment is recognized as crucial, its characterization in radiomics has often been limited to a single distance or coarse zoning, lacking a systematic multi-scale assessment that captures the spatial gradient of tumor invasion.^11^^,^^12^ Moreover, such models frequently lack integrated explainability tools and require further validation across truly independent, heterogeneous cohorts to confirm generalizability.

To address these limitations, we propose a methodological framework based on DCE-MRI for preoperative ALNM prediction. While the framework utilizes established computational techniques, its primary innovation lies in the systematic integration of multiscale characterization strategies. Specifically, we synergize fine-grained intratumoral habitat mapping via SLIC and K-means clustering with a systematic evaluation of peritumoral spatial gradients. By bridging these disparate analytical scales—intratumoral subregions, peritumoral microenvironments, and clinical profiles—this framework provides a more comprehensive “virtual biopsy” of the tumor ecosystem compared to existing coarse-grained models. Model interpretability is further enhanced with SHAP to ensure the mathematical outputs align with clinical radiological reasoning. A habitat-based segmentation strategy captures fine-grained intratumoral heterogeneity via superpixel clustering (SLIC) and K-means clustering. The peritumoral microenvironment is characterized using concentric rings at 1, 3, and 5 mm. From each habitat and peritumoral region, standardized, image biomarker standardization initiative (IBSI)-compliant radiomic features are extracted to develop the habitat signature and peritumoral signature, which are combined with clinical variables such as tumor size, histological grade, and receptor status. Feature selection and model development are conducted using least absolute shrinkage and selection operator (LASSO) and support vector machines (SVMs). Model interpretability is enhanced with Shapley additive explanations (SHAP), which decompose predictions into additive feature contributions, quantifying the impact of habitat and peritumoral signatures on ALNM risk. Robustness and generalizability are validated across multiple independent external cohorts. We hypothesize that this integrated, explainable framework may provide a reliable noninvasive tool for ALNM prediction and support personalized clinical decision-making in breast cancer.

## Results

### Clinical features

**Figure 1:**
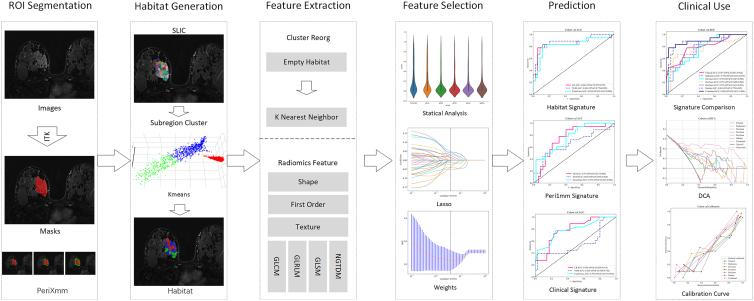
Overall workflow of this study

The overall flowchart of this study was displayed in Figure 1. We conducted a comprehensive univariate analysis of all clinical variables to assess their associations with the outcome, using odds ratios (ORs) and corresponding *p*-values. Variables with *p*-values less than 0.05 were considered statistically significant, suggesting their potential relevance as predictive factors. Baseline characteristics, including tumor location and BI-RADS category for all cohorts, are detailed in Table 1. As shown in Table 2, three clinical variables—tumor diameter, specific breast location (quadrant), and BI-RADS classification—were significantly associated with the outcome in both univariate and multivariate analyses (*p* < 0.05). These findings indicate that these variables serve as robust and independent predictors in the studied breast cancer cohort.

**Table 1:** Baseline characteristic of our cohorts

	Group				
Variable	Train, *N* =122^a^	Val, *N* =53^a^	Test1, *N* =99^a^	Test2, *N* =140^a^	*p* value^b^
Age, years	47.98±9.8	49.94±8.98	49.54±10.65	47.30±11.08	0.1
Diameter, mm	29.83±16.35	27.45±14.15	24.99±11.86	22.42±14.20	0.451
Location,n (%)					0.195
Upper inner	29(23.77)	15(28.30)	23(23.23)	35(25.00)	
Lower inner	21(17.21)	13(24.53)	15(15.15)	17(12.14)	
Outer upper	45(36.89)	17(32.08)	33(33.33)	66(47.14)	
Outer lower	27(22.13)	8(15.09)	28(28.28)	22(15.71)	
BI−RADS,n (%)					
≤4b	6(4.92)	8(15.09)	20(20.20)	21(15.00)	0.071
4c	11(9.02)	5(9.43)	23(23.23)	52(37.14)	
≥5	105(86.07)	40(75.47)	56(56.57)	67(47.86)	
Location2,n (%)					0.402
Left	63(51.64)	23(43.40)	48(48.48)	71(50.71)	
right	59(48.36)	30(56.60)	51(51.52)	69(49.29)	

a Mean±SD or Frequency (%).

b Kruskal−Wallis rank-sum test; Fisher’s exact test; Pearson’s Chi-squared test.

**Table 2:** Univariable and variable analysis of clinical features

Variable	OR_UNI	95% CI_UNI	*p*_value_UNI	OR_MULTI	95% CI_MULTI	*p*_value_MULTI
age	0.996	0.9890−1.0030	0.312	−	−	−
location2	1.008	0.8900−1.1420	0.92	−	−	−
diameter	1.014	1.0100−1.0170	0	1.012	1.0080−1.0150	0
location	1.114	1.0520−1.1790	0.002	1.069	1.0150−1.1250	0.037
BI_RADS	1.231	1.1190−1.3540	0	1.157	1.0600−1.2640	0.007

### Handcrafted features

**Figure 2:**
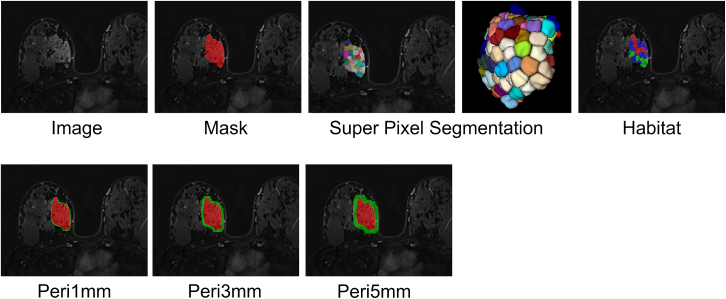
Generation of peritumoral and intratumoral heterogeneity regions

**Figure 3:**
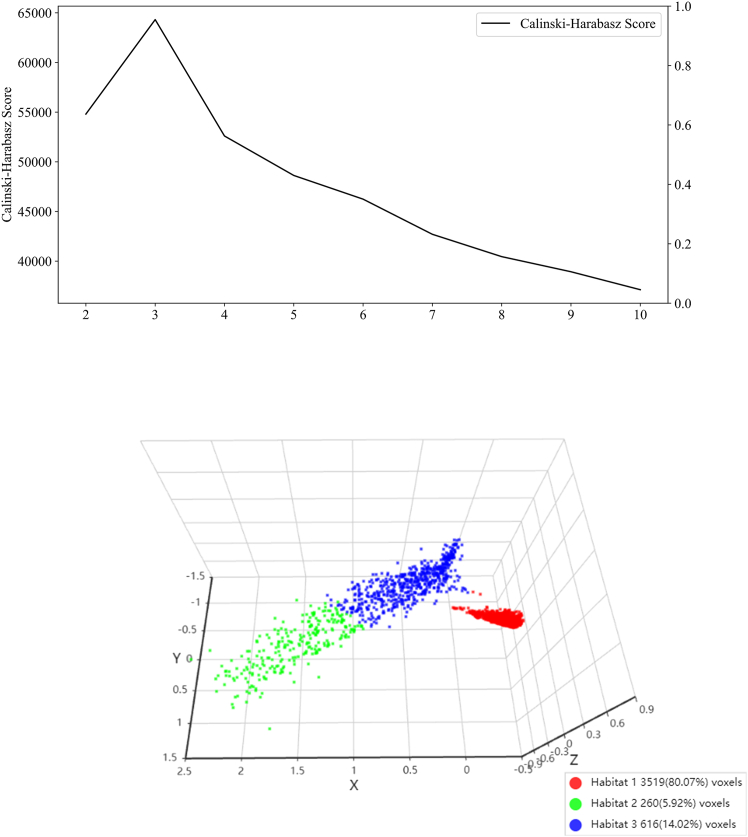
Evaluation of clustering index and habitat segmentation (A) Calinski-Harabasz (CH) index values for different numbers of clusters, with the highest score observed at K = 3, indicating the optimal number of clusters. (B) Habitat segmentation map generated based on K = 3, showing three distinct intratumoral subregions.

The process of tumor ROI extraction, peritumoral ring construction, and intratumoral habitat segmentation is illustrated in Figure 2. The optimal number of clusters for habitat segmentation was determined by evaluating values of K ranging from 2 to 10 and selecting the value that maximized the Calinski-Harabasz (CH) score. As shown in Figure 3A, the CH score reached its peak at K = 3, indicating the best trade-off between inter-cluster separation and intra-cluster compactness. Figure 3B illustrates the spatial distribution of the three identified habitat regions, demonstrating the effectiveness of the method in delineating distinct microenvironmental subzones within the tumor.

**Figure 4:**
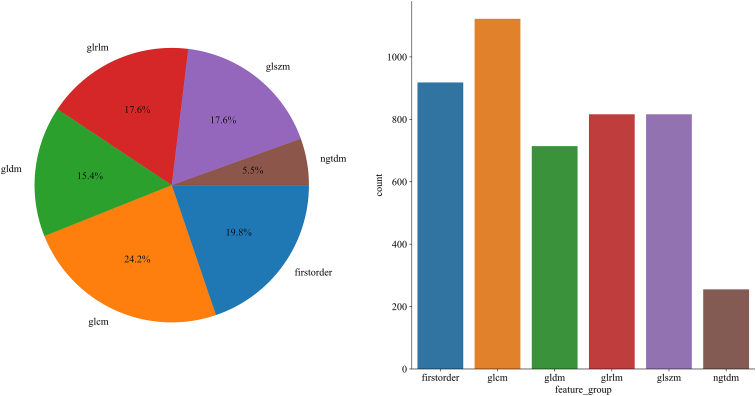
Distribution and composition of handcrafted radiomic features across different feature groups

For each segmented subregion, 1,547 handcrafted radiomic features were extracted, encompassing shape descriptors, first-order statistics, and texture-based metrics. As shown in Figure 4, 306 features were derived from first-order intensity distributions, 14 from shape-based descriptors, and the remaining features from texture families, including the gray-level co-occurrence matrix (GLCM), gray-level run-length matrix (GLRLM), gray-level size zone matrix (GLSZM), and neighboring gray-tone difference matrix (NGTDM). The habitat signature was constructed by aggregating features from all three clustered regions across two imaging modalities, resulting in a total of 4,641 features. For comparison, both the conventional Radiomics signature (whole-tumor) and the peritumoral signature consisted of 1,547 features each. All feature extraction procedures were performed using a custom pipeline developed with PyRadiomics. A detailed breakdown of the feature categories is provided in a supplementary figure, offering a comprehensive overview of the feature composition.

**Figure 5:**
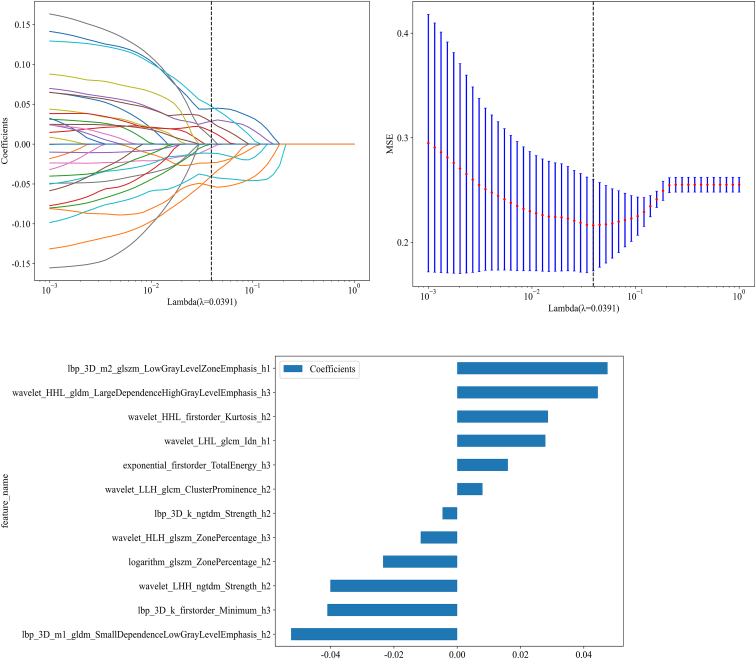
Feature selection and Rad-score distribution (A) Coefficients from 10-fold cross-validation, (B) MSE across 10-fold cross-validation, (C) histogram of the Rad-score based on selected features.

Least absolute shrinkage and selection operator (LASSO) logistic regression (LR) was applied to identify features with nonzero coefficients for Rad-score construction. As shown in Figure 5, the selected feature coefficients are displayed alongside the mean squared error (MSE) obtained through 10-fold cross-validation.

### Habitat signature

**Figure 6:**
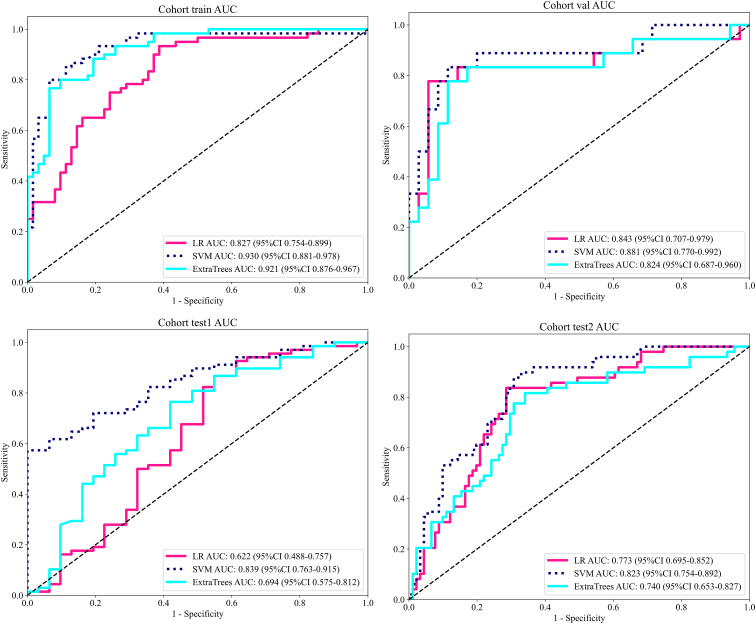
ROC curves of different models in each cohort

As presented in Table 3 and Figure 6, the support vector machine (SVM) model consistently demonstrated superior performance across all cohorts. In the training and internal validation cohorts, SVM achieved the highest AUC values of 0.930 (95% CI: 0.881–0.978) and 0.881 (95% CI: 0.770–0.992), respectively, outperforming both LR and ExtraTrees classifiers within the Habitat model framework. For the external test cohorts, SVM further exhibited superior performance, with AUC of 0.839 (95% CI: 0.763–0.915) in test1 and 0.823 (95% CI: 0.754–0.892) in test2. Notably, the SVM model maintained perfect specificity (1.000) in test1 and achieved the highest sensitivity (0.878) among all models in test2. These findings indicate that the SVM classifier offers the most balanced and robust performance, consistently demonstrating strong discriminative ability and generalizability. The robust AUC values, particularly in external validations, underscore the model’s potential clinical utility for predicting lymph node metastasis based on the proposed radiomic signatures. Detailed results of the radiomic and peritumoral feature analyses are provided in Supplementary materials 2.

**Table 3:** Model performance of different machine learning algorithms in each cohorts

Model	Accuracy	AUC	95% CI	Sensitivity	Specificity	PPV	NPV	Cohort
LR	0.770	0.827	0.754 - 0.899	0.933	0.613	0.700	0.905	train
LR	0.887	0.843	0.707 - 0.979	0.778	0.943	0.875	0.892	val
LR	0.737	0.622	0.488 - 0.757	0.868	0.452	0.776	0.609	test1
LR	0.757	0.773	0.695 - 0.852	0.837	0.714	0.612	0.890	test2
SVM	0.869	0.930	0.881 - 0.978	0.867	0.871	0.867	0.871	train
SVM	0.868	0.881	0.770 - 0.992	0.833	0.886	0.789	0.912	val
SVM	0.707	0.839	0.763 - 0.915	0.574	1.000	1.000	0.517	test1
SVM	0.757	0.823	0.754 - 0.892	0.878	0.692	0.606	0.913	test2
ExtraTrees	0.852	0.921	0.876 - 0.967	0.800	0.903	0.889	0.824	train
ExtraTrees	0.849	0.824	0.687 - 0.960	0.778	0.886	0.778	0.886	val
ExtraTrees	0.707	0.694	0.575 - 0.812	0.765	0.581	0.800	0.529	test1
ExtraTrees	0.714	0.740	0.653 - 0.827	0.816	0.659	0.563	0.870	test2

### SHAP value

**Figure 7:**
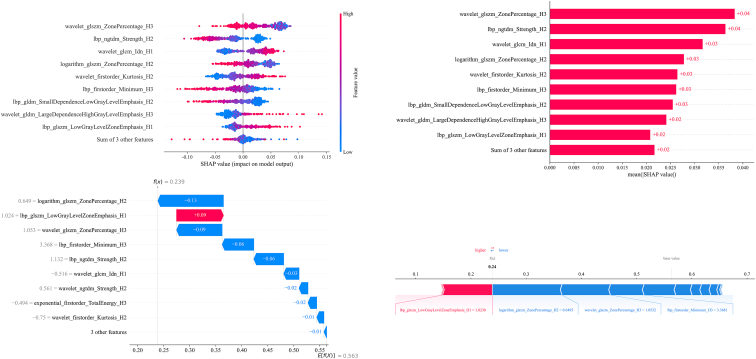
Visualization of feature contributions using SHAP values

SHAP analysis was performed on the SVM-based Habitat model to interpret feature contributions (Figure 7). To further enhance clinical interpretability, four representative cases were analyzed (Figure S10). In the true positive (TP) case, the prediction was primarily driven by H1 habitat features which capture specific heterogeneity associated with metastasis. Conversely, in the false positive (FP) and False Negative (FN) cases, the model’s errors were often linked to overlapping texture characteristics within the H2 and H3 habitats. Specifically, in FP cases, benign heterogeneity in these subregions could mimic metastatic patterns, while in FN cases, certain habitat features might dilute the positive signals from the H1 zone, leading to an underestimation of risk.

### Signature comparison

**Figure 8:**
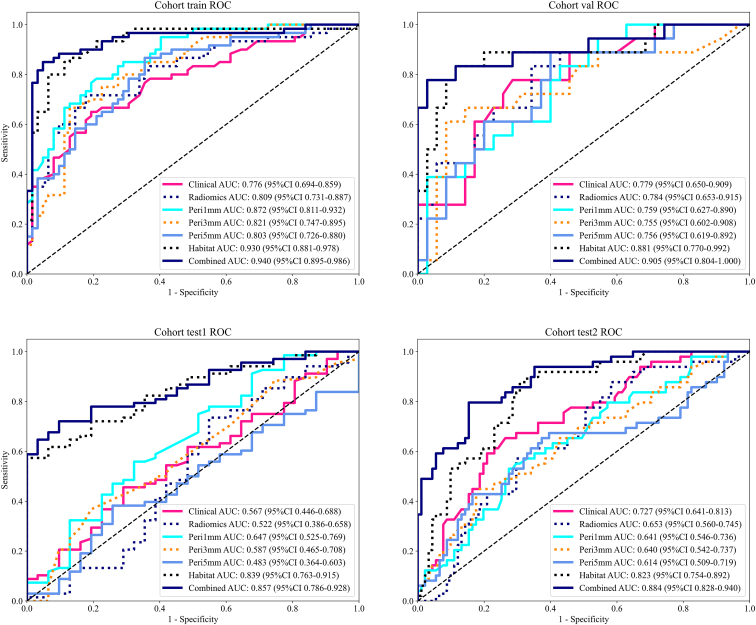
Receiver operating characteristic (ROC) curves of different predictive models across all cohorts

Across all cohorts, the habitat model consistently achieved the highest AUC values, outperforming both the conventional radiomics model and peritumoral models (Table 4; Figure 8). Specifically, in the training set, the habitat model achieved an AUC of 0.930 (95% CI: 0.881–0.978), compared to 0.872 (95% CI: 0.811–0.932) for the Peri1mm peritumoral model and 0.809 (95% CI: 0.731–0.887) for the radiomics model. Similar trends were observed in the validation cohort (AUC: 0.881 vs. 0.759 vs. 0.784) and the external test cohorts: test1 (AUC: 0.839 vs. 0.647 vs. 0.522) and test2 (AUC: 0.823 vs. 0.641 vs. 0.653). Among the peritumoral models, Peri1mm exhibited the best performance across all datasets. Furthermore, the combined model, integrating the habitat signature, Peri1mm peritumoral signature, and clinical variables, improved classification performance, achieving the highest AUCs of 0.940 (95% CI: 0.895–0.986) in training, 0.905 (95% CI: 0.804–1.000) in validation, 0.857 (95% CI: 0.786–0.928) in test1, and 0.884 (95% CI: 0.828–0.940) in test2. These results demonstrate the superiority of the habitat-based model over traditional radiomics and peritumoral approaches. Notably, Peri1mm provided more informative features than other peritumoral expansions. By integrating habitat, Peri1mm, and clinical variables, the combined model captured complementary intra- and peritumoral information, as well as patient-specific factors, leading to significantly improved predictive performance and enhanced generalizability across independent test cohorts.

**Table 4:** Model performance of different machine learning algorithms in each cohorts

Signature	Accuracy	AUC	95% CI	Sensitivity	Specificity	PPV	NPV	Cohort
Clinical	0.730	0.776	0.6943 - 0.8587	0.650	0.806	0.765	0.704	train
Radiomics	0.770	0.809	0.7307 - 0.8865	0.717	0.823	0.796	0.750	train
Peri1mm	0.787	0.872	0.8113 - 0.9317	0.783	0.790	0.783	0.790	train
Peri3mm	0.770	0.821	0.7466 - 0.8953	0.667	0.871	0.833	0.730	train
Peri5mm	0.754	0.803	0.7256 - 0.8803	0.867	0.645	0.703	0.833	train
Habitat	0.869	0.930	0.8814 - 0.9780	0.867	0.871	0.867	0.871	train
Combined	0.902	0.940	0.8950 - 0.9857	0.850	0.952	0.944	0.868	train
Clinical	0.736	0.779	0.6500 - 0.9088	0.778	0.714	0.583	0.862	val
Radiomics	0.717	0.784	0.6531 - 0.9152	0.833	0.657	0.556	0.885	val
Peri1mm	0.660	0.759	0.6270 - 0.8904	0.833	0.571	0.500	0.870	val
Peri3mm	0.811	0.755	0.6015 - 0.9080	0.611	0.914	0.786	0.821	val
Peri5mm	0.698	0.756	0.6191 - 0.8921	0.889	0.600	0.533	0.913	val
Habitat	0.868	0.881	0.7704 - 0.9915	0.833	0.886	0.789	0.912	val
Combined	0.906	0.905	0.8040 - 1.0000	0.778	0.971	0.933	0.895	val
Clinical	0.535	0.567	0.4462 - 0.6876	0.456	0.710	0.775	0.373	test1
Radiomics	0.646	0.522	0.3857 - 0.6579	0.735	0.452	0.746	0.437	test1
Peri1mm	0.596	0.647	0.5249 - 0.7692	0.559	0.677	0.792	0.412	test1
Peri3mm	0.505	0.587	0.4654 - 0.7082	0.368	0.806	0.806	0.368	test1
Peri5mm	0.495	0.483	0.3637 - 0.6031	0.382	0.742	0.765	0.354	test1
Habitat	0.707	0.839	0.7634 - 0.9150	0.574	1.000	1.000	0.517	test1
Combined	0.778	0.857	0.7860 - 0.9275	0.721	0.903	0.942	0.596	test1
Clinical	0.707	0.727	0.6406 - 0.8133	0.653	0.736	0.571	0.798	test2
Radiomics	0.579	0.653	0.5602 - 0.7455	0.878	0.418	0.448	0.864	test2
Peri1mm	0.657	0.641	0.5460 - 0.7364	0.531	0.725	0.510	0.742	test2
Peri3mm	0.686	0.640	0.5421 - 0.7371	0.449	0.813	0.564	0.733	test2
Peri5mm	0.636	0.614	0.5093 - 0.7192	0.653	0.626	0.485	0.770	test2
Habitat	0.757	0.823	0.7539 - 0.8918	0.878	0.692	0.606	0.913	test2
Combined	0.829	0.884	0.8279 - 0.9397	0.796	0.846	0.736	0.885	test2

### Calibration curve analysis

**Figure 9:**
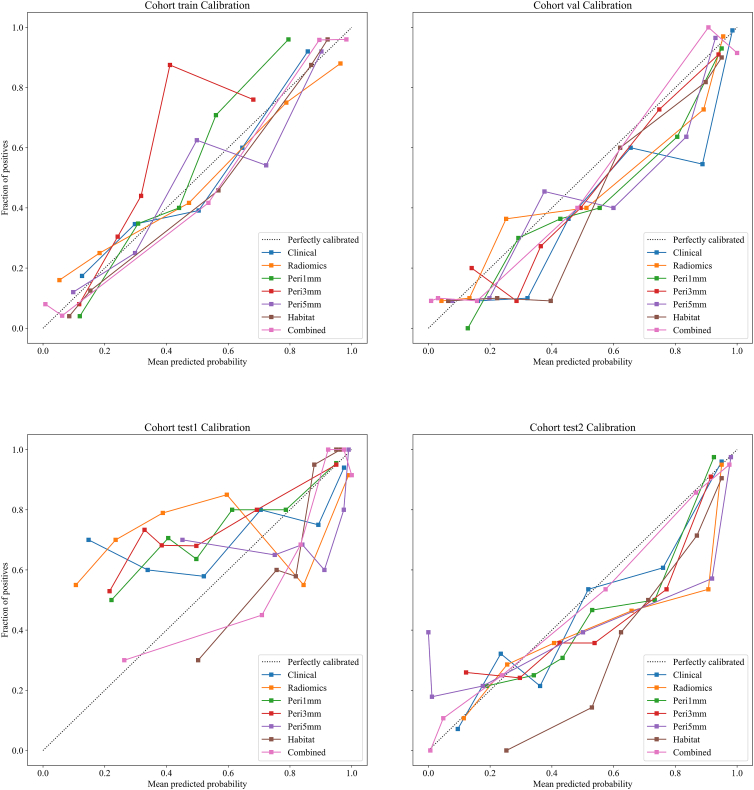
Calibration curves of different predictive models across all cohorts

Model calibration was assessed using the Hosmer-Lemeshow (HL) test, where lower HL values indicate better agreement between predicted probabilities and actual outcomes (Figure 9). The HL statistics for the training, validation, test1, and test2 cohorts were 0.225, 0.170, 0.100, and 0.346, respectively, indicating good calibration performance across all datasets.

### DeLong test

**Figure 10:**
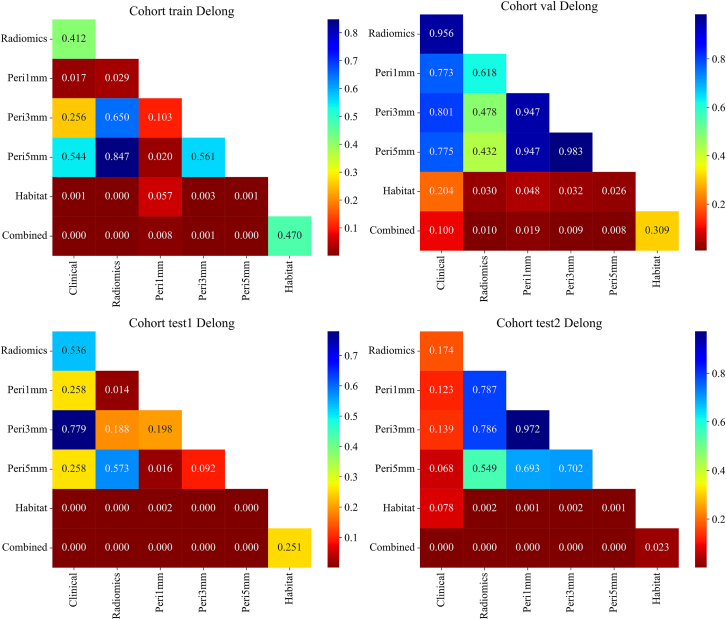
Pairwise comparison of signature performance using DeLong’s Test

DeLong pairwise AUC comparisons demonstrated that the combined model achieved statistically significant improvements compared to the clinical model, the conventional radiomics model, and the peritumoral models in both external test cohorts (Figure 10). Specifically, for both test1 and test2 cohorts, all comparisons involving the combined model yielded *p* < 0.05, confirming that the integration of habitat, 1 mm peritumoral, and clinical features resulted in an appreciable and reproducible improvement in discriminatory performance.

### Decision curve analysis and nomogram

**Figure 11:**
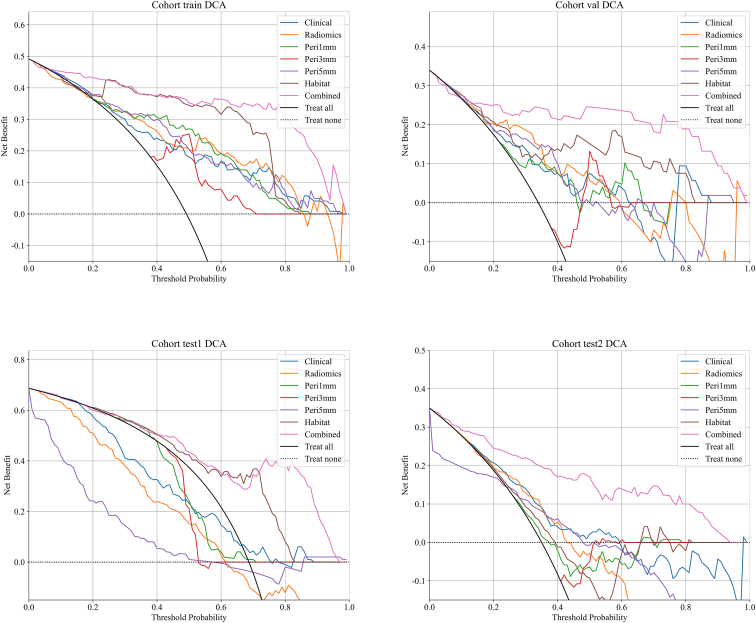
Decision curve analysis for evaluating net benefit across cohorts. Decision curves are shown for training, validation, test1, and test2 cohorts

**Figure 12:**
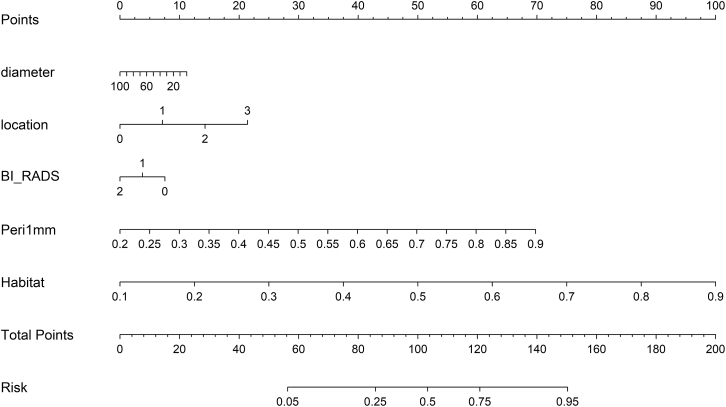
Clinical nomogram for prediction of lymph node metastasis

Decision curve analysis (DCA) curves for both the training and testing cohorts are presented in Figure 11. The analysis of these curves indicated that the combined model demonstrated a notable advantage in net benefit based on its predicted probabilities. Additionally, a nomogram was employed to visualize the results of the combined model (Figure 12).

## Discussion

In this study, our Habitat model exhibited strong predictive performance for (ALNM, achieving AUCs above 0.82 (0.839 [95% CI: 0.763–0.915] and 0.823 [95% CI: 0.754–0.892]) in two independent external cohorts, thereby demonstrating good generalizability—a common challenge in radiomics. SHAP analysis revealed that features from specific intratumoral “habitat” subregions were the most influential, highlighting the importance of modeling fine-grained tumor heterogeneity. These results are consistent with previous studies showing that habitat-based radiomics, which partitions tumors into subregions with distinct imaging characteristics, can effectively capture internal complexity and improve classification performance.^10^^,^^13^^,^^14^ Chen et al. demonstrated that MRI-based habitat analysis exhibited excellent performance in identifying key tumor characteristics, with reported AUC values ranging from 0.81 to 0.94.^15^ Similarly, SHAP was applied to radiomics and topological features derived from fused PET/CT images to enhance the interpretability of prognostic models, identifying key features such as RadScore as significant predictors in the framework.^16^ In our study, SHAP visualizations provided spatially resolved insights, indicating that texture features from specific habitat regions—particularly between the tumor core and periphery—contributed most significantly to prediction. These findings highlight the added value of our habitat-based approach, which moves beyond the conventional radiomics model (whole-tumor analysis) by pinpointing biologically relevant subregions associated with metastatic potential.

To quantify the independent contributions of the various feature groups, we conducted a systematic comparison as summarized in Table 4. In addition to intratumoral heterogeneity, incorporating multiscale peritumoral information and clinical variables significantly enhanced model performance. Our analysis identifies the Habitat signature as the primary driver of the model, exhibiting robust standalone performance with AUCs exceeding 0.82 in both external cohorts. In contrast, the clinical and conventional radiomics signatures showed significantly lower independent discriminative power. Among the peritumoral configurations, the 1 mm expansion (Peri1mm) yielded the most discriminative performance. Crucially, the integration of these complementary components in the combined model provided a significant incremental benefit; for instance, it achieved the peak AUC of 0.884 (95% CI: 0.828–0.940) in external test cohort 2, compared to 0.823 (95% CI: 0.754–0.892) for the habitat signature alone. This hierarchical improvement underscores how habitat features capture essential tumor biology, while peritumoral and clinical data provide the necessary micro- and macro-environmental context to maximize generalizability. Among these, the 1 mm peritumoral expansion (Peri1mm) yielded the most discriminative performance across all cohorts, underscoring the added value of fine-scale peritumoral information. This finding is biologically plausible, given the critical role of the tumor microenvironment in invasion and metastasis. Our results are consistent with previous studies supporting the value of peritumoral characterization.^17^^,^^18^^,^^19^ For example, in DCE-MRI, adding peritumoral features within a 4 mm radius improved model performance, with AUC up to 0.877.^20^ Similarly, integrated intra- and peritumoral models have shown strong predictive ability, achieving AUC of 0.916 and 0.764 in the training and validation cohorts, respectively.^21^ Our findings indicate that peritumoral rings with a 1 mm expansion yielded the highest predictive performance, suggesting that this mid-range distance is optimal for extracting informative peritumoral characteristics. Although minor AUC variations were noted between our two external cohorts—likely due to differences in imaging protocols or patient demographics—the consistently high performance reinforces the robustness of our combined model.

Compared with existing ALN prediction models, our approach offers both methodological innovation and competitive performance. While previous studies—such as that by Huang et al.—have reported high accuracy using whole-tumor DCE-MRI features, combined model provides superior discriminative ability while incorporating more biologically informed spatial information.^22^ Specifically, we employed superpixel-based clustering followed by K-means to define tumor habitats, enabling detailed characterization of intratumoral regions. By integrating intra-habitat features, multiscale peritumoral characteristics, and clinical variables, and leveraging LASSO for feature selection and machine learning for classification, our model capitalizes on complementary data sources.

Clinically, our combined model offers a noninvasive decision-support tool for preoperative axillary staging. Both ALN dissection and SLNB carry risks of morbidity, including lymphedema and nerve injury.^6^^,^^23^ A reliable, interpretable model can help identify low-risk patients who may safely avoid invasive procedures, thus reducing overtreatment and improving quality of life.^24^^,^^25^ As reported by Liu et al., MRI-based nomograms could potentially spare unnecessary axillary interventions in node-negative patients.^26^ Our model could complement conventional imaging by serving as a “virtual biopsy” of the tumor and its surrounding microenvironment. As illustrated by the representative cases in Figure S10, SHAP-based explanations enhance clinical interpretability by highlighting the most influential tumor habitats and peritumoral zones.^27^ This interpretability translates into practical clinical utility by providing a transparent “reasoning guide” that supplements the model’s probability score. For example, by identifying the specific texture features in the H1 habitat that drive a high-risk prediction, the model allows radiologists to cross-verify the radiomic signature with morphological imaging patterns. Furthermore, in cases where the model yields a False Positive, SHAP plots can reveal whether the error was induced by confounding factors like peritumoral inflammation. Such insights empower clinicians to critically evaluate the model’s output in the context of individual patient biology. Overall, our habitat-based, multiscale radiomics model serves as an “interpretable virtual biopsy” that can help identify low-risk candidates for surgical de-escalation, thereby minimizing overtreatment while optimizing preoperative axillary staging.

It is important to emphasize that while the computational components of our pipeline—such as SLIC superpixels, K-means clustering, and SVM—are well-established in the literature; the primary innovation of this study lies in their synergistic methodological integration into a robust multiscale framework. Unlike many previous radiomics models that lack stability when applied to external data, our framework maintained a high and consistent level of performance across three independent centers with heterogeneous imaging protocols. This multi-center robustness serves as an empirical “proof of concept” that the strategic combination of intratumoral habitat mapping and peritumoral spatial gradients provides a more comprehensive and generalizable characterization of tumor biology than the individual techniques could achieve in isolation. Consequently, the value of this work resides in the development of a reliable, clinically validated diagnostic tool that overcomes the common challenges of overfitting and poor generalizability in high-dimensional radiomic analysis.

In summary, we have proposed an integrated methodological framework based on DCE-MRI that synergizes the habitat signature, multiscale peritumoral signature, and clinical variables within an interpretable combined model to predict ALN metastasis. The promising results on external validation cohorts suggest that it may serve as a valuable preoperative adjunct, though confirmation through large-scale, prospective, multicenter studies with fully automated workflows is a critical next step.

### Limitations of the study

Despite the encouraging performance, several limitations should be acknowledged. First, this retrospective study was conducted at a limited number of centers, and prospective, large-scale validation across diverse populations and imaging platforms is required to confirm generalizability. Second, tumor regions were manually segmented by expert radiologists, which is labor-intensive and may introduce inter-observer variability, potentially limiting scalability in routine clinical practice. Third, although the model incorporates explainable techniques, radiomic features remain indirectly related to underlying tumor biology, and the absence of spatially matched genomic or histopathological data precludes definitive biological interpretation of the identified habitats. Finally, the current framework has not yet been prospectively integrated into clinical workflows, and its real-world impact on surgical decision-making remains to be established.

## Resource availability

### Lead contact

Requests for further information and resources should be directed to and will be fulfilled by the lead contact, Zhenfeng Huang (huangzhf28@mail.sysu.edu.cn).

### Materials availability

This study did not generate new unique reagents.

### Data and code availability

• The radiological images and clinical data in this study are not publicly available due to patient privacy concerns. However, these data may be available from the lead contact upon reasonable request and with appropriate data use agreements. The code supporting the findings of this study has been deposited in the GitHub repository and is publicly available under the DOI: <u>https://github.com/OnekeyAI-Platform/huangzhf7. https://github.com/OnekeyAI-Platform/huangzhf7 given here and in subsequent occurrences is invalid or inaccessible. Please verify the address and correct if required.
• Any additional information required to reanalyze the data reported in this article is available from the lead contact upon request.

## Acknowledgments

This research received no external funding. We thank the participating hospitals and clinical staff for their support in data collection and management.

## Author contributions

J.D., Z.Q., and H.P. contributed equally to this work and are considered co-first authors. J.D.: conceptualization, methodology, and writing – original draft; Z.Q.: data curation, formal analysis, and visualization; H.P.: data curation and validation; S.C.: data curation; Q.Z.: investigation and project administration; Y.Z.: supervision, writing – review and editing; Z.Y.: conceptualization and project administration; Z.H.: writing – review, resources, and funding acquisition. Z.H., Z.Y., and Y.Z. are co-corresponding authors.

## Declaration of interests

The authors declare no competing interests.

## Declaration of generative AI and AI-assisted technologies in the writing process

During the preparation of this manuscript, ChatGPT was used solely for language editing and improving clarity. All content was subsequently reviewed and revised by the authors, who take full responsibility for the accuracy and integrity of the work.

## STAR★Methods

### Key resources table

**Table:** 

REAGENT or RESOURCE	SOURCE	IDENTIFIER
**Deposited data**		

Code for models	This paper	https://github.com/OnekeyAI-Platform/huangzhf7
Clinical data of patients	This paper	N/A
MRI images	This paper	N/A

**Software and algorithms**		

ITK-SNAP (v3.8.0)	ITK-SNAP Development Core Team	https://www.itksnap.org/
Python 3.8	Python Development Core Team	https://www.python.org/
SPSS 26.0	Stanford University	https://www.ibm.com/products/spss-statistics

### Experimental model and study participant details

#### Ethics statement

This retrospective analysis has received approval from the ethics committees of the Fifth Affiliated Hospital of Sun Yat-sen University (SYSU Hospital; approval number: K230-1), Huizhou Central People’s Hospital (HZ Hospital; approval number: ky112024007), and Linyi City People Hospital (LY Hospital; approval number: 202402-H-003). Informed consent was waived due to the retrospective nature of the study, which was conducted in accordance with the Declaration of Helsinki.

#### Participants

A total of 414 female patients diagnosed with primary invasive breast cancer between 2021 and 2024 at the three participating centers were screened for inclusion. The mean age of the included participants was 48.4 ± 10.3 years, with an overall age range of 28 to 79 years. Following the journal’s reporting standards, all participants enrolled in this study were individuals of East Asian descent, Chinese, and Han nationality. Eligibility criteria required: (1) pathologically confirmed primary invasive breast cancer via surgery or biopsy; (2) presence of a solitary, solid tumor; (3) availability of pre-treatment breast MRI including DCE-MRI performed within two weeks prior to surgery or biopsy; and (4) no prior neoadjuvant chemotherapy or other anti-tumor interventions. Patients were excluded based on: (1) any treatment prior to imaging; (2) concurrent malignancies at other sites; (3) incomplete clinical, imaging, or pathological data; or (4) poor MRI image quality. To rigorously assess model robustness, the 414 patients were divided into three distinct cohorts: 175 patients from Huizhou Central People’s Hospital (HZ Hospital) were randomly allocated into a training cohort (n=122) and an internal validation set (n=53) in a 7:3 ratio using stratified sampling to maintain balanced distributions of clinical variables; 99 patients from the Fifth Affiliated Hospital of Sun Yat-sen University (SYSU Hospital) served as independent external validation cohort 1; and 140 patients from Linyi City People Hospital (LY Hospital) served as independent external validation cohort 2. Baseline characteristics, including tumor location and BI-RADS category for all cohorts, are detailed in Table 1. Regarding the influence of sex and gender on the study results, as this research focuses exclusively on female breast cancer, only female participants were included (n=414, 100% female), and thus the influence of sex could not be separately analyzed.

As this study focuses exclusively on female breast cancer, only female participants were included (n=414, 100% female). Therefore, the influence of sex and gender on the results could not be analyzed, which is acknowledged as a limitation of this study.

### Method details

#### Image acquisition and preprocessing

All MRI scans were performed using standard clinical protocols at the participating institutions. To minimize inter-scanner variability and standardize spatial resolution, all DCE-MRI volumes were resampled to an isotropic voxel size of 1 × 1 × 1 mm^3^. Image intensities were normalized using trilinear interpolation, and the corresponding tumor segmentation masks were resampled using nearest-neighbor interpolation to preserve label integrity. Additionally, the N4 bias field correction method was applied to correct non-uniform magnetic field effects and improve intensity homogeneity across scans.

#### Tumor and peritumoral region segmentation

Tumor segmentation and region-of-interest (ROI) construction were conducted to enable detailed analysis of both intratumoral heterogeneity and the peritumoral microenvironment. Manual delineation of the primary tumor was performed on axial DCE-MRI slices using ITK-SNAP software by two experienced radiologists (with 5 and 8 years of experience, respectively). To ensure the spatial consistency and quality of the ground truth, the independent segmentations from both readers were compared across the entire dataset using the Dice Similarity Coefficient (DSC). Any case with a DSC below 0.85 was systematically flagged and subsequently adjudicated by a senior radiologist (with >20 years of experience) to reach a final consensus. This adjudicated consensus segmentation served as the definitive mask for all subsequent analyses, including habitat mapping and peritumoral region definition.

To model the peritumoral tissue environment, we applied a morphological dilation technique to expand the tumor mask outward at fixed distances of 1 mm, 3 mm, and 5 mm. Subtraction of the inner ROI yielded non-overlapping, ring-shaped peritumoral zones. These multiscale regions facilitated the systematic evaluation of spatial gradients in tissue characteristics surrounding the tumor. All peritumoral expansion steps were performed using the OnekeyAI mask-padding toolkit, which utilizes morphological dilation based on the SimpleITK library. Given that all images were resampled to a 1 mm^3^ isotropic resolution, dilation operations were set to 1, 3, and 5 pixels to achieve corresponding physical expansions. To ensure biological relevance, all generated peritumoral rings were visually inspected alongside the original MRI. Any portions of the rings extending into non-breast tissues or air were manually removed by the researchers using ITK-SNAP. This ensured that the analyzed regions exclusively represented the peritumoral breast parenchyma and its microenvironment.

Intratumoral heterogeneity was captured by partitioning each tumor ROI into approximately 100 spatially constrained superpixels using the Simple Linear Iterative Clustering (SLIC) algorithm—a granularity chosen to ensure a fine-grained initial subdivision while providing sufficient primitive regions for subsequent aggregation. Nineteen radiomic features were extracted from each superpixel. To ensure a reproducible and biologically consistent definition of subregions across the cohort, the optimal cluster number (K) was determined by pooling superpixel features from the training cohort (N=122) and maximizing the Calinski-Harabasz index, which yielded a peak at K=3 (Figure 3A). These training-derived cluster centroids were then used as a fixed template to map habitats in the validation and test cohorts. This joint-clustering and centroid-mapping approach ensures that the identified habitats maintain a standardized physical meaning and spatial coherence across all patients, facilitating robust and reproducible performance evaluation. Habitat maps were generated using the scikit-learn library (v1.0.2) and were visually reviewed for quality assurance (Figure 2 illustrates the tumor ROI, peritumoral ring construction, and intratumoral habitat segmentation process).

#### Radiomic feature extraction

Radiomic features were extracted separately from intratumoral habitats and peritumoral rings. Three categories of features were considered: (1) shape descriptors (e.g., volume, compactness, surface area); (2) first-order statistics (e.g., mean intensity, skewness, kurtosis); and (3) texture features derived from GLCM, GLRLM, GLSZM, and NGTDM matrices. For each clustered habitat subregion, features were extracted and aggregated to represent subregional phenotype. Missing values caused by small or homogeneous subregions were imputed using K-nearest neighbors (KNN). Feature extraction followed the Image Biomarker Standardization Initiative (IBSI) guidelines.

#### Feature selection

To reduce redundancy and strictly mitigate the risk of overfitting, especially given the high-dimensional nature of the initial 4,641 features, a multi-stage feature selection pipeline was applied. Initially, univariate filtering removed features lacking significant association with ALN status. Next, to mitigate multicollinearity, highly correlated feature pairs (Pearson |r| > 0.9) were identified. An iterative procedure was applied to remove redundancy: in each iteration, the feature with the largest number of high correlations (|r| > 0.9) to other features was eliminated until no such correlations remained in the feature set. The remaining features were ranked using the Minimum Redundancy Maximum Relevance (mRMR) algorithm, and the top 32 were retained. A final selection was performed using LASSO logistic regression with 10-fold cross-validation to identify non-zero coefficient features for model development.Ultimately, this pipeline refined the feature pool to a final set of 12 highly discriminative predictors. By maintaining a feature-to-sample ratio of approximately 1:10 (12 features for 122 training samples), we ensured that the model complexity was appropriate for the dataset size, thereby enhancing its robustness and generalizability.

#### Model development

Using the final selected features, we developed classification models for four signature types. Radiomics signatures were derived from whole-tumor area (Radiomics signature), peritumoral rings (Peritumoral signature). The Habitat model was constructed using a linear-kernel Support Vector Machine (SVM) and Light Gradient Boosting Machine (LightGBM) to capture different decision boundaries. For peritumoral zones, separate SVMs assessed the predictive contribution of each ring. The Habitat signature aggregated radiomic features from superpixel-clustered subregions, reflecting intratumoral heterogeneity.

A clinical signature was built from variables significant in univariate analysis (p < 0.05) using regularized logistic regression, with missing values imputed by mean or median. Finally, the Combined model integrated the Habitat signature, the Peri1mm Peritumoral signature, and clinical predictors via stepwise multivariate selection (entry p < 0.05). Hyperparameters were optimized with grid search and 5-fold cross-validation, and model performance was evaluated by AUC.

### Quantification and statistical analysis

The SPSS software (version 26) and Python was used to perform analysis. Normality of continuous variables was assessed using the Shapiro–Wilk test. Between-group comparisons were conducted using Student’s t-test or Mann–Whitney U test, as appropriate. Categorical variables were compared using the chi-squared test. Model discrimination was evaluated using ROC analysis, and metrics including AUC, accuracy, sensitivity, specificity, positive predictive value (PPV), and negative predictive value (NPV) were reported. Calibration was assessed using the Hosmer–Lemeshow test and calibration plots. Model performance comparisons were conducted using DeLong’s test.

## Supplemental information

Document S1. Figures S1−S10 and Tables S1−S5
