# Supplementary material for: Peritumoral habitat radiomics predicts axillary lymph node metastasis in breast cancer
Source: iScience. 2026 Mar 16;29(4):115365. doi: 10.1016/j.isci.2026.115365 (PMC13081050; doi:10.1016/j.isci.2026.115365)
Supplement: Document S1. Figures S1−S10 and Tables S1−S5 [file mmc1.pdf]

iScience, Volume *29*

Supplemental information

Peritumoral habitat radiomics predicts axillary lymph node metastasis in breast cancer

Jingwen Ding, Zhikun Qiu, Hongbin Peng, Shuyuan Chen, Qiaowei Zhong, Yideng Zhang, Zhiwei Yang, and Zhenfeng Huang

Supplemental information

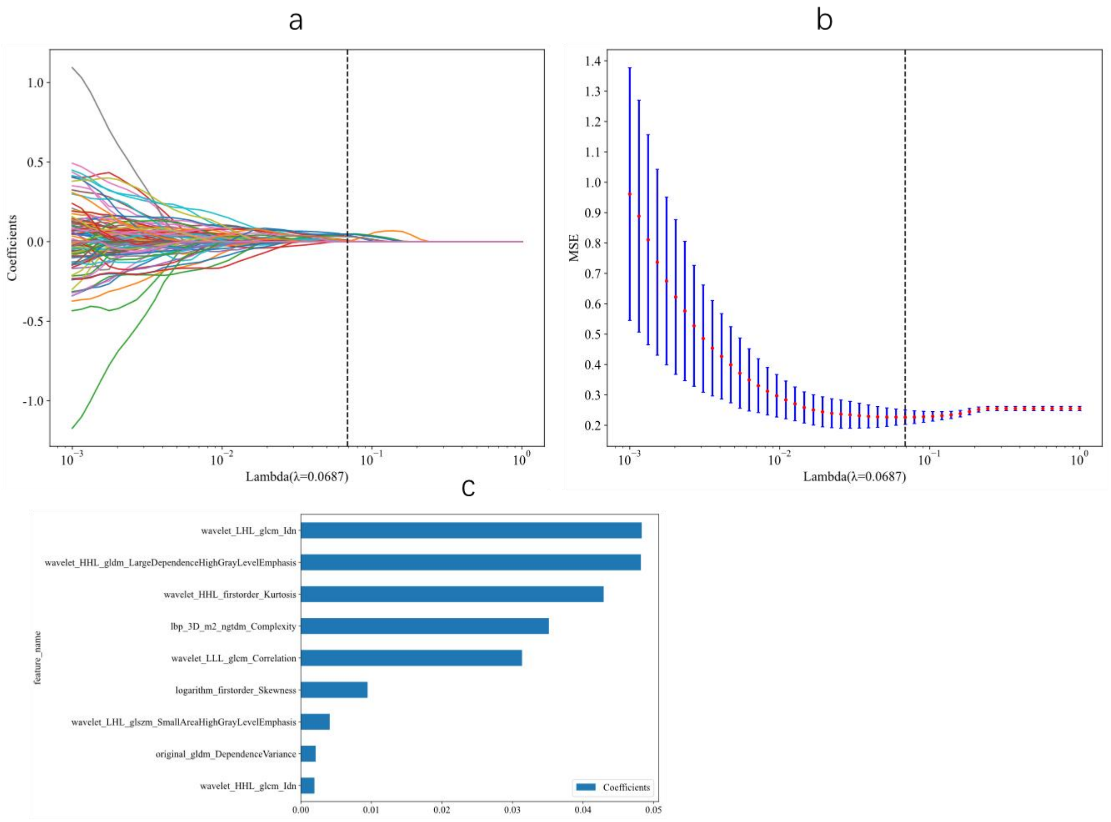

Figure S1. Radiomics feature selection for the intratumoral signature, Related to Figure 5. (a) Coefficients from 10-fold cross-validation, (b) MSE across 10-fold cross-validation, (c) Histogram of the Rad-score based on selected features.

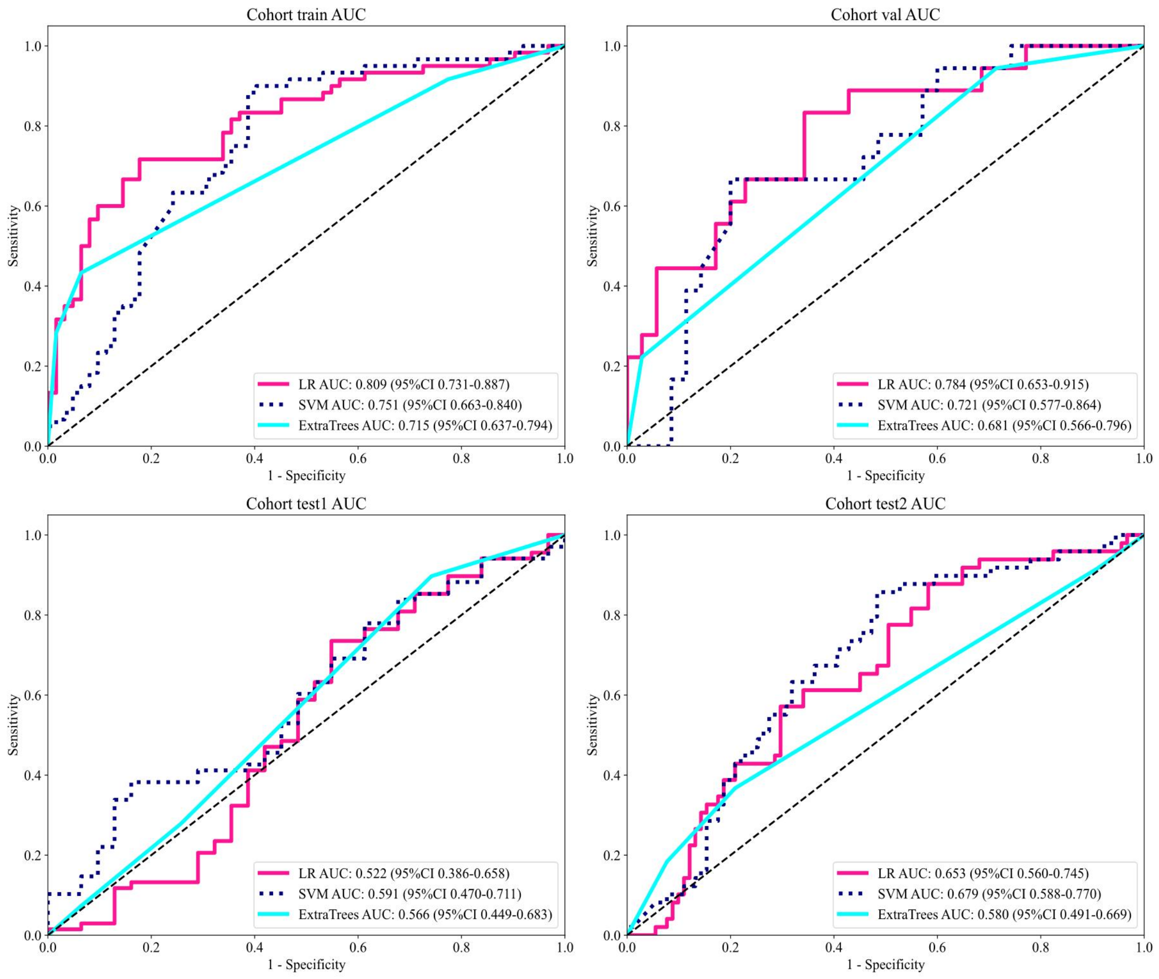

Figure S2. ROC curves of the intratumoral signature across cohorts, Related to Figure 6.

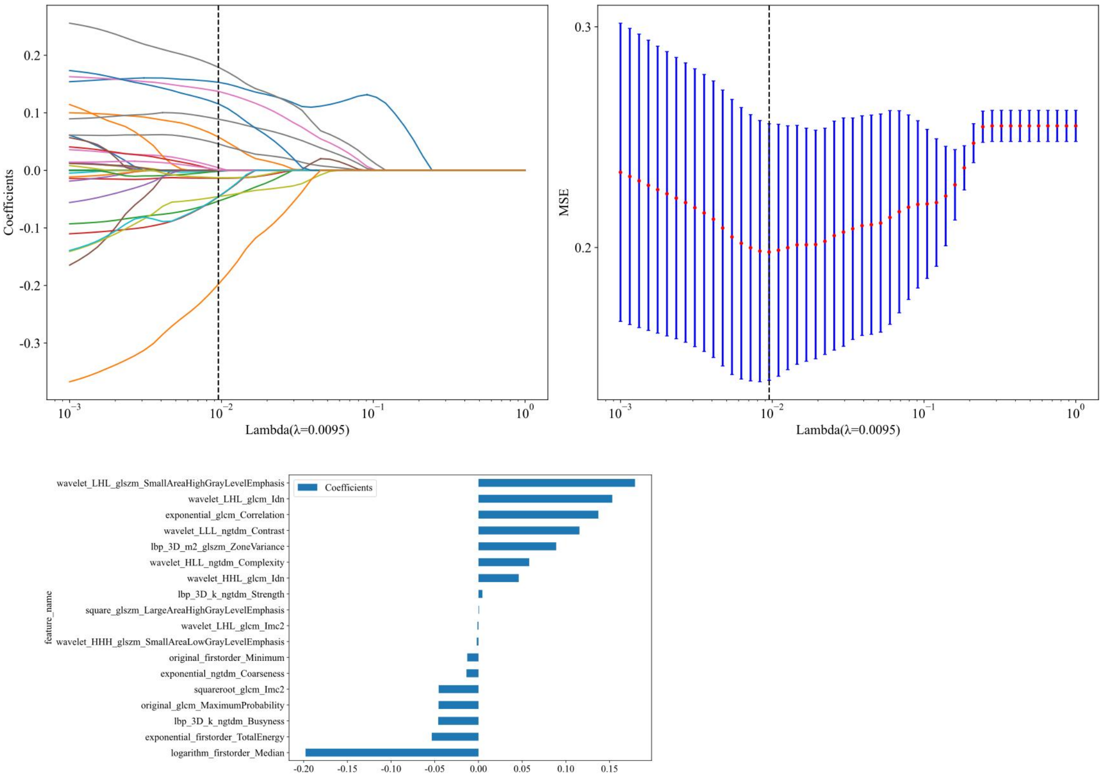

Figure S3. Radiomics feature selection for the peritumoral 1 mm signature, Related to Figure 8. (a) Coefficients from 10-fold cross-validation, (b) MSE across 10-fold cross-validation, (c) Histogram of the Rad-score based on selected features

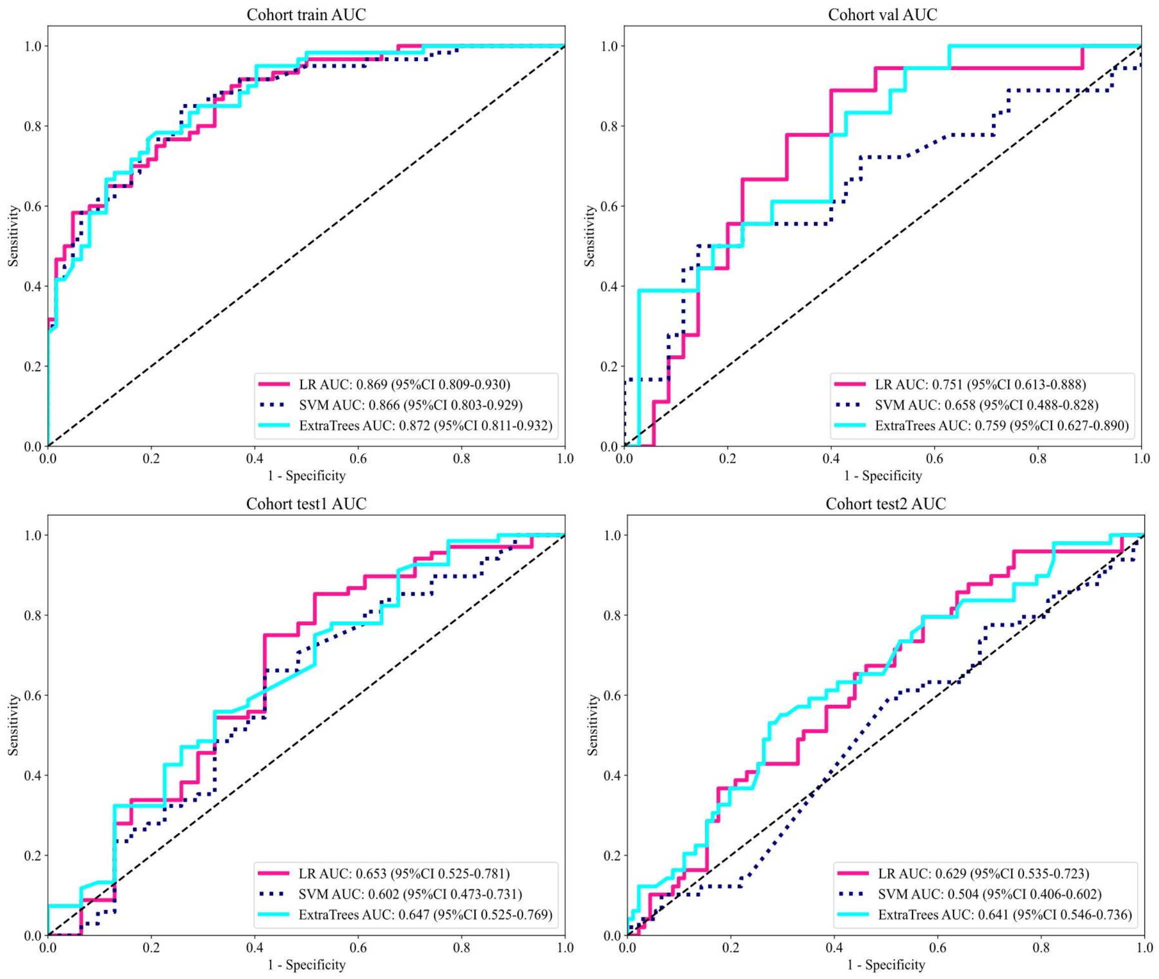

Figure S4. ROC curves of the peritumoral 1 mm signature, Related to Figure 8.

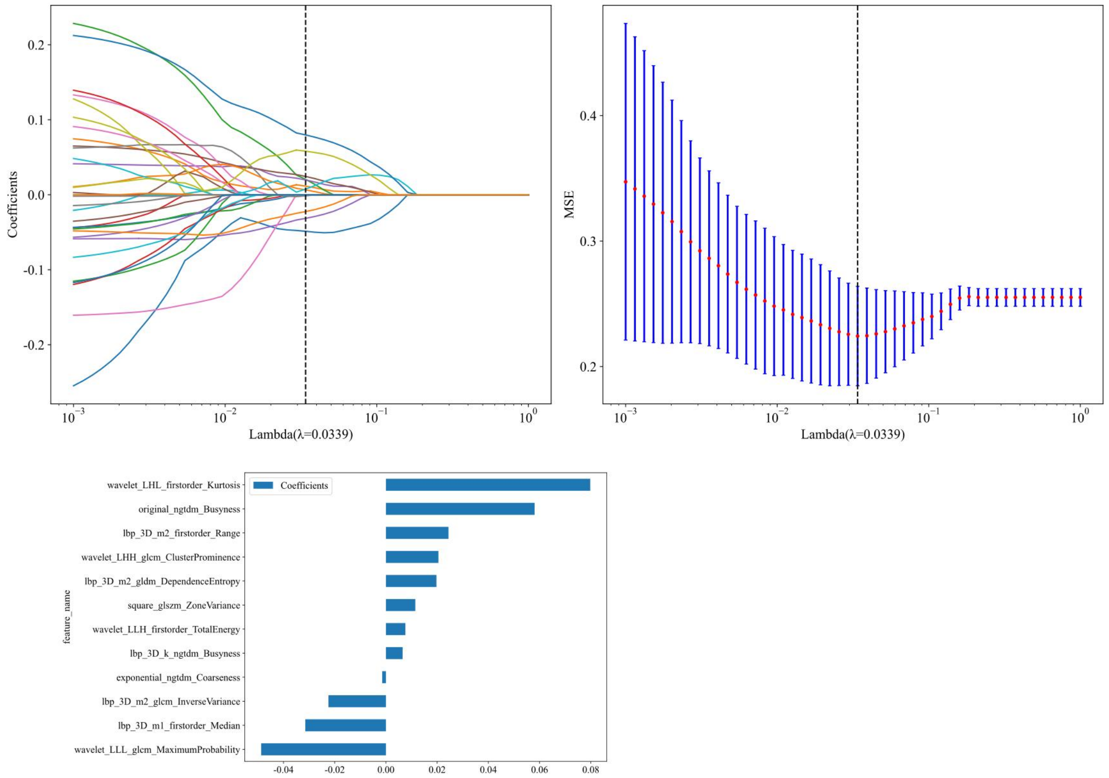

Figure S5. Radiomics feature selection for the peritumoral 3 mm signature, Related to Figure 8. (a) Coefficients from 10-fold cross-validation, (b) MSE across 10-fold cross-validation, (c) Histogram of the Rad-score based on selected features.

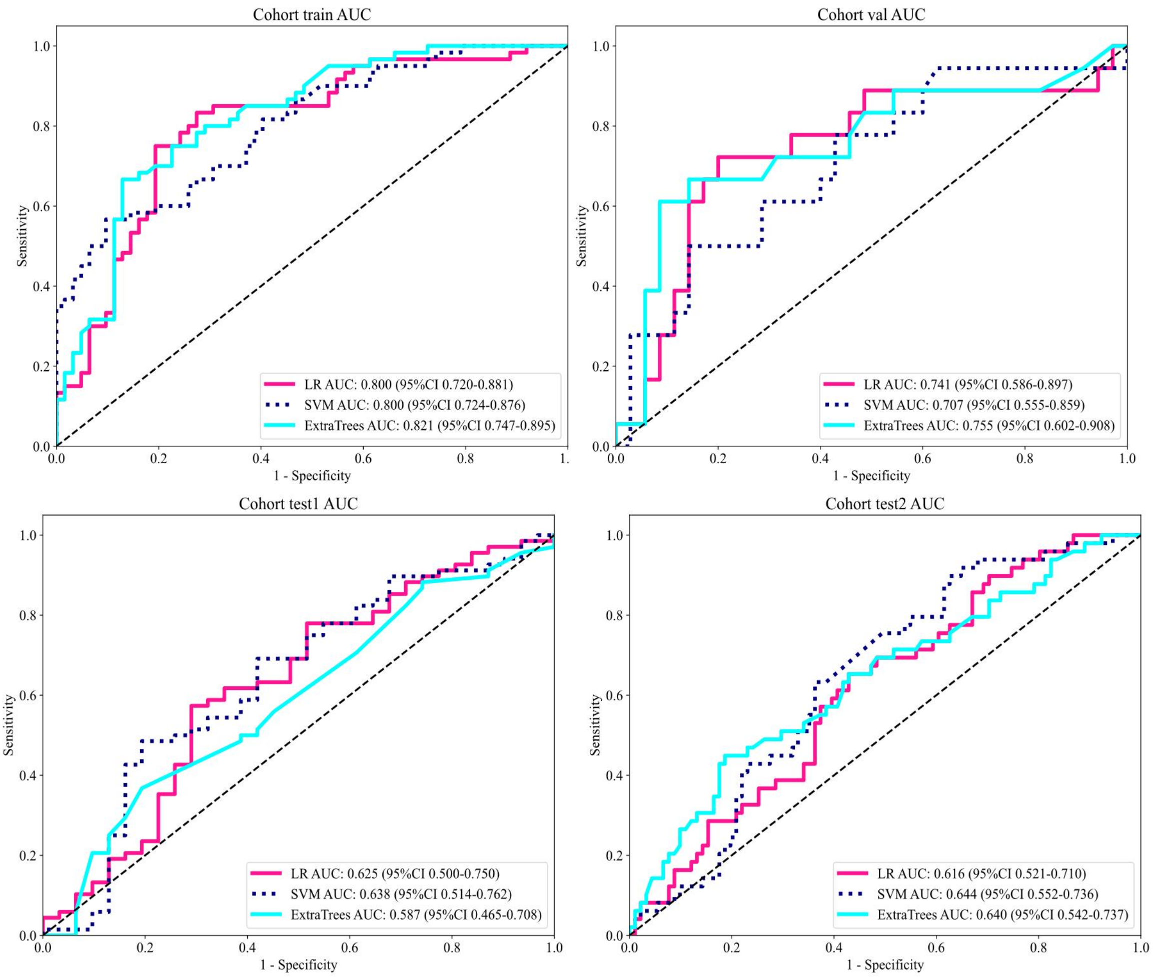

Figure S6. ROC curves of the peritumoral 3 mm signature, Related to Figure 8.

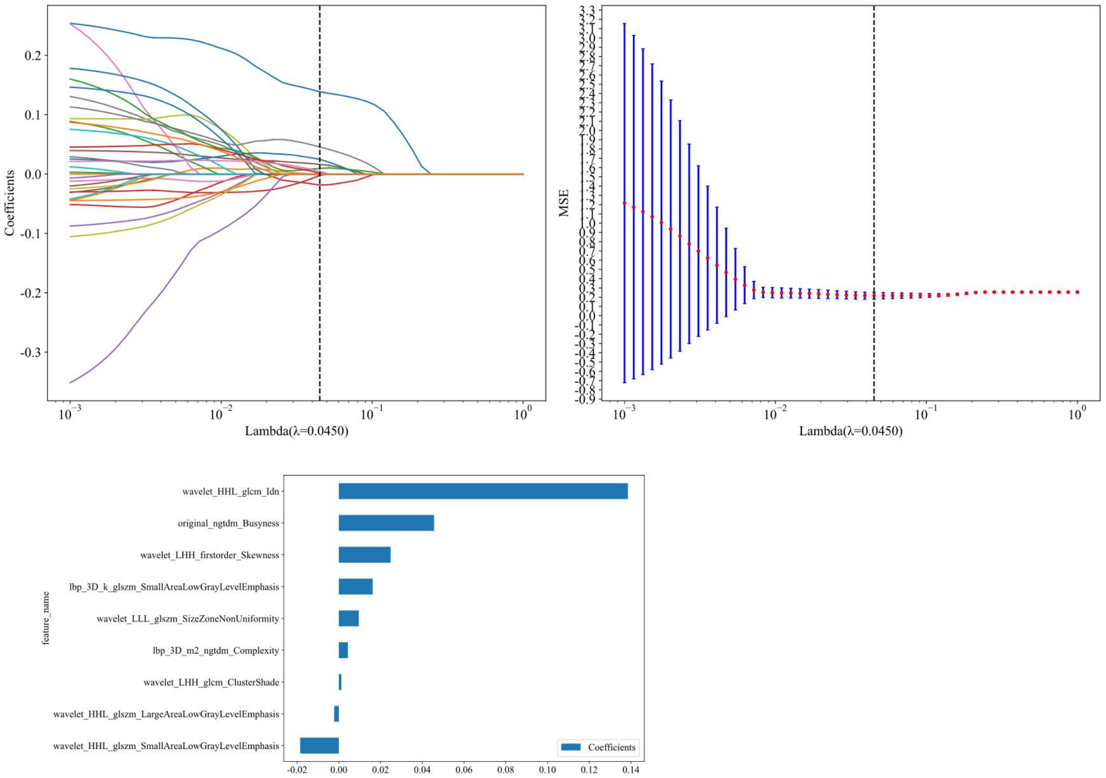

Figure S7. Radiomics feature selection for the peritumoral 5 mm signature, Related to Figure 8. (a) Coefficients from 10-fold cross-validation, (b) MSE across 10-fold cross-validation, (c) Histogram of the Rad-score based on selected features.

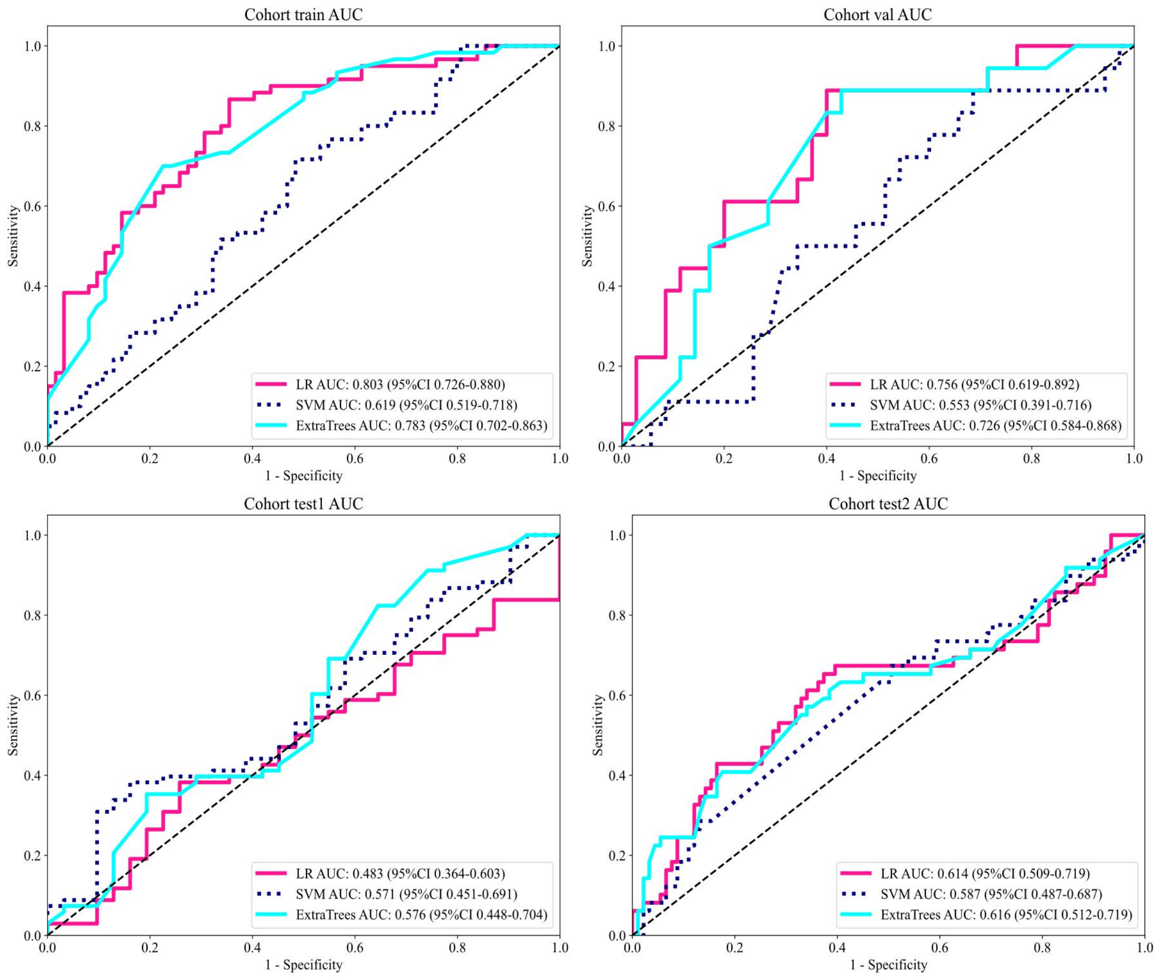

Figure S8. ROC curves of the peritumoral 5 mm signature, Related to Figure 8.

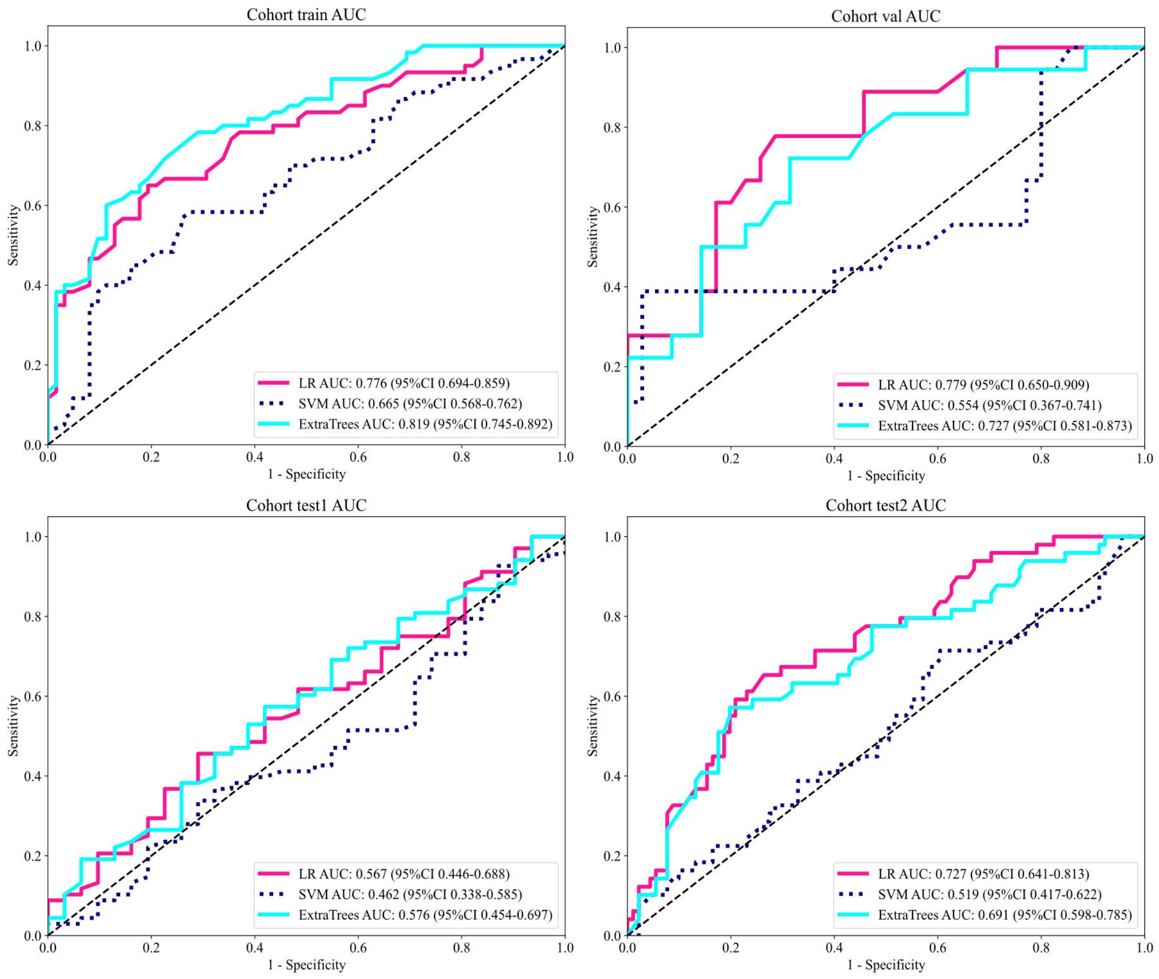

Figure S9. ROC curves of the clinical model, Related to Figure 8.

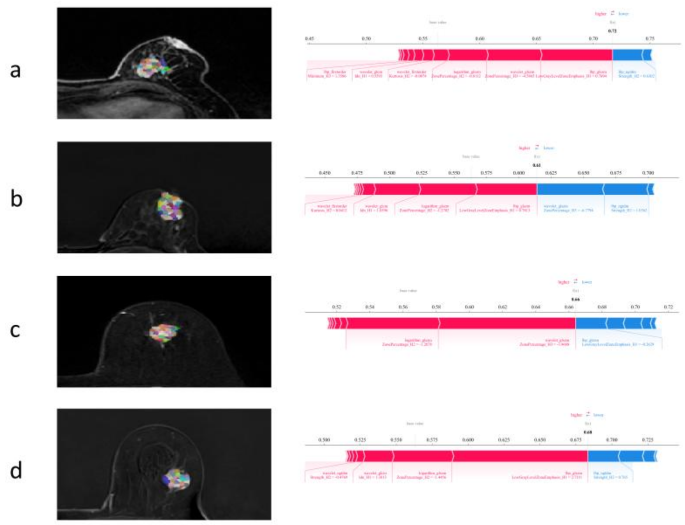

Figure S10. SHAP-based interpretability analysis of representative cases, Related to Figure 7. (a) True Positive case: Correct prediction of metastasis driven by high-risk features in the H1 habitat. (b) True Negative case: Correct prediction of non-metastasis with low risk-scores across subregions. (c) False Positive case: Overestimation of risk potentially due to benign heterogeneity in the H2 region. (d) False Negative case: Underestimation of risk where positive signals from the H1 habitat were offset by other subregional features.

Table S1. Model performance of the intratumoral signature across cohorts, Related to Figure 6.

Model	Accuracy	AUC	95% CI	Sensitivity	Specificity	PPV	NPV	Cohort
LR	0.770	0.809	0.731 - 0.887	0.717	0.823	0.796	0.750	train
LR	0.717	0.784	0.653 - 0.915	0.833	0.657	0.556	0.885	val
LR	0.646	0.522	0.386 - 0.658	0.735	0.452	0.746	0.437	test1
LR	0.579	0.653	0.560 - 0.745	0.878	0.418	0.448	0.864	test2
SVM	0.746	0.751	0.663 - 0.840	0.900	0.597	0.684	0.860	train
SVM	0.755	0.721	0.577 - 0.864	0.667	0.800	0.632	0.824	val
SVM	0.525	0.591	0.470 - 0.711	0.382	0.839	0.839	0.382	test1
SVM	0.636	0.679	0.588 - 0.770	0.857	0.516	0.488	0.870	test2
ExtraTrees	0.689	0.715	0.637 - 0.794	0.433	0.935	0.867	0.630	train
ExtraTrees	0.509	0.681	0.566 - 0.796	0.944	0.286	0.405	0.909	val
ExtraTrees	0.697	0.566	0.449 - 0.683	0.897	0.258	0.726	0.533	test1
ExtraTrees	0.643	0.580	0.491 - 0.669	0.367	0.791	0.486	0.699	test2

Table S2. Model performance of the peritumoral 1 mm signature, Related to Figure 8.

Model	Accuracy	AUC	95% CI	Sensitivity	Specificity	PPV	NPV	Cohort
LR	0.770	0.869	0.809 - 0.930	0.917	0.629	0.705	0.886	train
LR	0.698	0.751	0.613 - 0.888	0.889	0.600	0.533	0.913	val
LR	0.737	0.653	0.525 - 0.781	0.853	0.484	0.784	0.600	test1
LR	0.557	0.629	0.535 - 0.723	0.796	0.429	0.429	0.796	test2
SVM	0.795	0.866	0.803 - 0.929	0.850	0.742	0.761	0.836	train
SVM	0.736	0.658	0.488 - 0.828	0.500	0.857	0.643	0.769	val
SVM	0.636	0.602	0.473 - 0.731	0.662	0.581	0.776	0.439	test1
SVM	0.529	0.504	0.406 - 0.602	0.592	0.495	0.387	0.692	test2
ExtraTrees	0.787	0.872	0.811 - 0.932	0.783	0.790	0.783	0.790	train
ExtraTrees	0.660	0.759	0.627 - 0.890	0.833	0.571	0.500	0.870	val
ExtraTrees	0.596	0.647	0.525 - 0.769	0.559	0.677	0.792	0.412	test1
ExtraTrees	0.657	0.641	0.546 - 0.736	0.531	0.725	0.510	0.742	test2

Table S3. Model performance of the peritumoral 3 mm signature, Related to Figure 8.

Model	Accuracy	AUC	95% CI	Sensitivity	Specificity	PPV	NPV	Cohort
LR	0.779	0.800	0.720 - 0.881	0.833	0.726	0.746	0.818	train
LR	0.774	0.741	0.586 - 0.897	0.722	0.800	0.650	0.848	val
LR	0.616	0.625	0.500 - 0.750	0.574	0.710	0.812	0.431	test1
LR	0.600	0.616	0.521 - 0.710	0.653	0.571	0.451	0.754	test2
SVM	0.738	0.800	0.724 - 0.876	0.567	0.903	0.850	0.683	train
SVM	0.736	0.707	0.555 - 0.859	0.500	0.857	0.643	0.769	val
SVM	0.586	0.638	0.514 - 0.762	0.485	0.806	0.846	0.417	test1
SVM	0.557	0.644	0.552 - 0.736	0.898	0.374	0.436	0.872	test2
ExtraTrees	0.770	0.821	0.747 - 0.895	0.667	0.871	0.833	0.730	train
ExtraTrees	0.811	0.755	0.602 - 0.908	0.611	0.914	0.786	0.821	val
ExtraTrees	0.505	0.587	0.465 - 0.708	0.368	0.806	0.806	0.368	test1
ExtraTrees	0.686	0.640	0.542 - 0.737	0.449	0.813	0.564	0.733	test2

Table S4. Model performance of the peritumoral 5 mm signature, Related to Figure 8.

Model	Accuracy	AUC	95% CI	Sensitivity	Specificity	PPV	NPV	Cohort
LR	0.754	0.803	0.726 - 0.880	0.867	0.645	0.703	0.833	train
LR	0.698	0.756	0.619 - 0.892	0.889	0.600	0.533	0.913	val
LR	0.495	0.483	0.364 - 0.603	0.382	0.742	0.765	0.354	test1
LR	0.636	0.614	0.509 - 0.719	0.653	0.626	0.485	0.770	test2
SVM	0.615	0.619	0.519 - 0.718	0.717	0.516	0.589	0.653	train
SVM	0.509	0.553	0.391 - 0.716	0.889	0.314	0.400	0.846	val
SVM	0.525	0.571	0.451 - 0.691	0.382	0.839	0.839	0.382	test1
SVM	0.557	0.587	0.487 - 0.687	0.673	0.495	0.418	0.738	test2
ExtraTrees	0.738	0.783	0.702 - 0.863	0.700	0.774	0.750	0.727	train
ExtraTrees	0.679	0.726	0.584 - 0.868	0.889	0.571	0.516	0.909	val
ExtraTrees	0.677	0.576	0.448 - 0.704	0.824	0.355	0.737	0.478	test1
ExtraTrees	0.679	0.616	0.512 - 0.719	0.408	0.824	0.556	0.721	test2

Table S5. Model performance of the clinical model across cohorts, Related to Figure 8.

Model	Accuracy	AUC	95% CI	Sensitivity	Specificity	PPV	NPV	Cohort
LR	0.689	0.776	0.694 - 0.859	0.717	0.661	0.672	0.707	train
LR	0.736	0.779	0.650 - 0.909	0.722	0.743	0.591	0.839	val
LR	0.545	0.567	0.446 - 0.688	0.529	0.581	0.735	0.360	test1
LR	0.671	0.727	0.641 - 0.813	0.673	0.670	0.524	0.792	test2
SVM	0.656	0.665	0.568 - 0.762	0.567	0.742	0.680	0.639	train
SVM	0.623	0.554	0.367 - 0.741	0.389	0.743	0.437	0.703	val
SVM	0.455	0.462	0.338 - 0.585	0.368	0.645	0.694	0.317	test1
SVM	0.493	0.519	0.417 - 0.622	0.449	0.516	0.333	0.635	test2
ExtraTrees	0.730	0.819	0.745 - 0.892	0.783	0.677	0.701	0.764	train
ExtraTrees	0.698	0.727	0.581 - 0.873	0.722	0.686	0.542	0.828	val
ExtraTrees	0.495	0.576	0.454 - 0.697	0.412	0.677	0.737	0.344	test1
ExtraTrees	0.686	0.691	0.598 - 0.785	0.592	0.736	0.547	0.770	test2
